# Non-permissive human conventional CD1c^+^ dendritic cells enable *trans*-infection of human primary renal tubular epithelial cells and protect BK polyomavirus from neutralization

**DOI:** 10.1371/journal.ppat.1009042

**Published:** 2021-02-16

**Authors:** Mathieu Sikorski, Flora Coulon, Cécile Peltier, Cécile Braudeau, Alexandra Garcia, Matthieu Giraud, Karine Renaudin, Christine Kandel-Aznar, Steven Nedellec, Philippe Hulin, Julien Branchereau, Joëlle Véziers, Pauline Gaboriaud, Antoine Touzé, Julien Burlaud-Gaillard, Régis Josien, Dorian McIlroy, Céline Bressollette-Bodin, Franck Halary

**Affiliations:** 1 Nantes Université, Inserm, CHU Nantes, Center for Research in Transplantation and Immunology UMR1064, ITUN, Nantes, France; 2 CHU Nantes, Laboratoire d’Immunologie, CIMNA, Nantes, France; 3 CHU Nantes, Service d’Anatomie et Cytologie Pathologiques, Nantes, France; 4 MicroPicell imaging facility, Structure Fédérative de Recherche Santé François Bonamy—FED 4203/UMS Inserm 016/CNRS 3556, Nantes, France; 5 CHU Nantes, Service d’urologie, Nantes, France; 6 CHU Nantes, Service de transplantations rénales, Nantes, France; 7 INSERM, UMRS 1229, RMeS, Université de Nantes, ONIRIS, Nantes, France; 8 CHU Nantes, PHU4 OTONN, Nantes, France; 9 INSERM, UMS 016, CNRS 3556, Structure Fédérative de Recherche François Bonamy, SC3M facility, Université de Nantes, Nantes, France; 10 Université de Nantes, UFR Odontologie, Nantes, France; 11 Infectiologie et Santé Publique, UMR INRAE 1282, UFR de Sciences Pharmaceutiques, Université de Tours, Tours, France; 12 Département des Microscopies, Plateforme IBiSA de Microscopie Electronique, Université de Tours, Tours, France; 13 CHU Nantes, Laboratoire de virologie, Nantes, France; Brown University, UNITED STATES

## Abstract

The BK polyomavirus (BKPyV) is a ubiquitous human virus that persists in the renourinary epithelium. Immunosuppression can lead to BKPyV reactivation in the first year post-transplantation in kidney transplant recipients (KTRs) and hematopoietic stem cell transplant recipients. In KTRs, persistent DNAemia has been correlated to the occurrence of polyomavirus-associated nephropathy (PVAN) that can lead to graft loss if not properly controlled. Based on recent observations that conventional dendritic cells (cDCs) specifically infiltrate PVAN lesions, we hypothesized that those cells could play a role in BKPyV infection. We first demonstrated that monocyte-derived dendritic cells (MDDCs), an *in vitro* model for mDCs, captured BKPyV particles through an unconventional GRAF-1 endocytic pathway. Neither BKPyV particles nor BKPyV-infected cells were shown to activate MDDCs. Endocytosed virions were efficiently transmitted to permissive cells and protected from the antibody-mediated neutralization. Finally, we demonstrated that freshly isolated CD1c^+^ mDCs from the blood and kidney parenchyma behaved similarly to MDDCs thus extending our results to cells of clinical relevance. This study sheds light on a potential unprecedented CD1c^+^ mDC involvement in the BKPyV infection as a promoter of viral spreading.

## Author summary

Dr Sylvia Gardner first discovered the BK polyomavirus (BKPyV) in the urine of a kidney-transplant recipient in 1970. In the 1990’s, the widespread use of potent immunosuppressive drugs such as tacrolimus, sirolimus or mycophenolate mofetil led to the emergence of BKPyV nephropathy. Recently, various studies reported a specific influx of myeloid dendritic cells (mDCs) in the renal tissue of kidney-transplant patients who were diagnosed with a BKPyV nephropathy. MDCs are immune cells both residing in tissues and migrating to other organs or compartments like the blood when changes in their environment occur. Their main functions are the detection of danger signals such as pathogens or tumors and the processing of antigens to prime naïve specific effectors of the adaptive immune response. Although anti-BKPyV cellular immune responses have been investigated in post-transplant recipients as well as healthy individuals, supporting an active role of mDCs little is known about how mDCs and BKPyV interact with each other. Our study provides the basis to understand the role played by mDCs in virus capture through an unprecedented endocytic mechanism and possibly in viral protection from neutralization by specific antibodies. Moreover, we showed that mDCs are unable to sense BKPyV particles or BKPyV-infected dying cells as a danger signal, supporting the view that other DC subsets might act as the true antigen presenting cells that promote the adaptive immune response against BKPyV infection.

## Introduction

The BK polyomavirus (BKPyV) is a small non-enveloped DNA virus. Its icosahedral capsid is mainly composed of the major capsid protein VP1[1–3]. Its prevalence in the worldwide population ranges from 80 to 90%[4,5]. Asymptomatic primary infection mostly occurs during childhood[6,7] followed by a persistent infection in the renourinary epithelium[8]. Although evidence of BKPyV reactivation was reported in kidney and hematopoietic stem cell allografts[9–12], it has been well established that BKPyV, reactivating in KTRs, is mainly of donor origin[13–17]. Viral shedding in urine possibly progressing to BKPyV-DNAemia first marks reactivation. Persistent BKPyV-DNAemia above 10^4^ DNA copies/ml has been correlated to PVAN (overall 1–5% of KTRs)(18–20). To date, BKPyV remains a significant cause of kidney graft failure[11,18].

Over the last ten years, anti-BKPyV cellular and humoral immune responses have been investigated demonstrating a prominent role of both specific CD4^+^ and CD8^+^ cytotoxic T lymphocytes (CTLs), mainly recognizing the large T antigen (LTAg)- and VP1-derived peptides associated with various HLA molecules[19–22]. Although anti-BKPyV responses are likely to be protective enough in healthy individuals, only ten percent of those shed virions in urine suggesting a limited impact of escape mechanisms[5]. DCs are known to orchestrate anti-viral immune responses mainly through their ability to cross-present viral antigens (Ag), thus efficiently priming or activating naïve or memory specific T cells, respectively[23]. To date, anti-polyomavirus (PyV) CTL responses in mice and humans were analyzed on autologous PBMCs or DCs stimulation using viral peptide pools thus bypassing the requirement for Ag processing, including endocytosis, and presentation by HLA class I molecules[20,23,24]. Only few studies addressed the ability of PyV to bind to, promote maturation or infect DCs. In mice, Drake and colleagues showed that splenic DCs are activated following infection by a murine PyV (MuPyV) strain thus allowing them to prime a CTL response[24]. Using another experimental setup, Lenz and colleagues demonstrated that although HPV16, a carcinogenic papillomavirus, and bovine papillomavirus virus-like particles (VLPs) enabled bone marrow-derived DC maturation, BKPyV or JCPyV VLPs did not[25]. More recently, hamster PyV (HaPyV)- and Trichodysplasia Spinulosa-associated PyV-derived VLPs were shown to moderately activate murine splenocytes[26]. Similarly, SV40 was shown to infect and activate MDDCs from rhesus macaques[27]. Human MDDCs were shown to support β-propiolactone-inactivated BKPyV-derived Ag presentation while remaining unresponsive to native BK- and JCPyV particles[28] possibly due to distinct viral Ag processing induced by inactivation[29]. Gedvilaite and colleagues also reported that human MDDCs were responsive to MuPyV and HaPyV VLPs[28]. Mostly, DCs, although limited to *in vitro* generated cells, seemed to be unresponsive to BK- or JC PyV direct exposure and poorly responsive to BKPyV-derived Ags in KTRs and immunocompetent individuals, as recently proposed by Kaur et al[30]. The mechanisms behind such DC unresponsiveness remain to be explored regardless of the presence of immunosuppressive drugs.

In the healthy kidney, cDC, including the CD1c^+^ DC subset, are located within the interstitium[31], close to the renal proximal tubular epithelial cells (hRPTECs), a host cell for BKPyV[32]. HRPTECs were shown to negatively regulate cDC activation subsequently leading to the retention of cDCs in renal tissues as immature cells[33–35] putatively decreasing Ag presentation by DCs. Early stage PVAN is marked by a CD1c^+^ cDC infiltrate[36] and mild inflammation[32,37,38]. Whether cDCs play a role in the pathophysiology of the BKPyV infection apart from their ability to trigger and sustain specific immune responses is still unclear.

Here, we demonstrate for the first time that myeloid DC, i.e., MDDCs and freshly isolated CD1c^+^ cDC from the blood and the kidney of healthy donors, but not plasmacytoid DC were capable of capturing BKPyV particles through the CLIC/GEEC endocytic pathway and transmitting them to hRPTECs without getting activated or infected. We also showed that endocytosed BKPyV particles were protected from antibody-mediated neutralization offering to cDC subsets the possibility to participate in BKPyV spreading in the kidney at least in early steps of the reactivation.

## Results

### Human monocyte-derived dendritic cells bind BKPyV particles in a dose- and sialic acid-dependent manner

**Fig 1:**
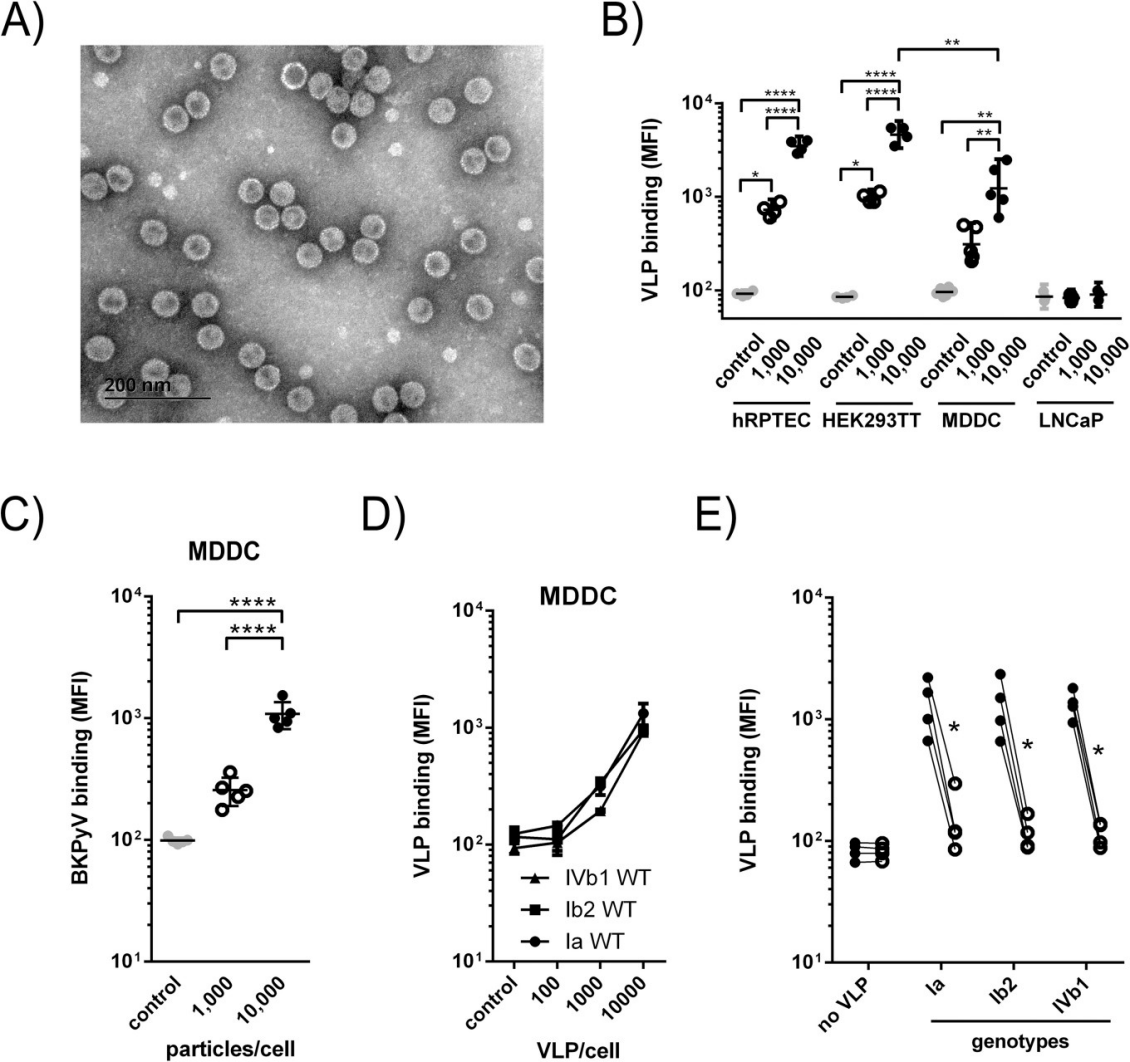
MDDCs bind BKPyV particles in a dose- and sialic acid-dependent manner. (A) Negative contrast TEM picture of genotype Ib2 (gIb2) VLPs. A 200nm scale bar is represented on the micrograph. (B) Fluorescent-labelled gIb2 VLP binding to hRPTECs, HEK293TT, MDDCs and LNCaP cells (n = 3) assessed by flow cytometry. Mean Fluorescence Intensities (MFIs) are displayed (n = 5; n = 3 for LNCaP only). (C) Alexa Fluor 647-conjugated infectious particles (Dunlop strain) binding to MDDCs (n = 5). (D) Dose dependent binding of genotypes Ia (circle), Ib2 (square) and IVb1 (triangle) VLPs to MDDCs (n = 5). (E) Alexa Fluor 647-conjugated genotypes Ia, Ib2 and IVb1 VLPs (10^4^ VLPs/cell) binding to MDDCs with (empty circles) or without (closed circles) treatment with 0.2U/mL neuraminidase from *Clostridium perfringens*, specifically cleaving α(2,3/6/8)-linked sialic acid. Data are represented as MFI ± SEM. Statistically significant results were marked by one or several asterisks according to the level of significance: * = p<0.05, ** = p<0.01, **** = p<0.0001; one-way ANOVA with Tukey’s multiple comparison tests.

First, we assessed whether MDDCs could bind BKPyV particles using fluorescent-labelled genotype Ib2 (gIb2) BKPyV VLPs. VLP integrity was checked by negative contrast TEM (Fig 1A). GIb2 VLP binding was then tested with two BKPyV permissive cell types, namely hRPTECs and HEK293TT but also to MDDCs and LNCaP cells, a BKPyV non-permissive prostatic cancer cell line[39] at various VLPs/cell ratios. MDDCs effectively bound gIb2 VLPs in a dose-dependent manner but to a lesser extent compared to hRPTECs or HEK293TT (Fig 1B), and as expected BKPyV particles were unable to attach to LNCaP cells. MDDCs were also shown to bind gIa infectious particles (Fig 1C, Dunlop strain; [40]) and genotypes Ia, Ib2 and IVb1 VLPs had similar binding properties to MDDCs (Fig 1D). Sialic acids decorating *b*-series gangliosides are known as crucial components for BKPyV infection of hRPTECs and HEK293TT[39]. Then, we demonstrated that when MDDCs are treated with an appropriate neuraminidase, an enzyme known to specifically remove sialic acid moieties from the plasma membrane (PM), gIa, gIb2 and gIVb1 VLP binding was strongly impaired (Fig 1E). Altogether, these results clearly established that MDDCs could bind BKPyV from the most frequent genotypes in Europe and Asia in a dose and sialic acid-dependent manner.

### BKPyV particles are endocytosed in pleiomorphous tubular and macropinosome-like endosomes in MDDCs

**Fig 2:**
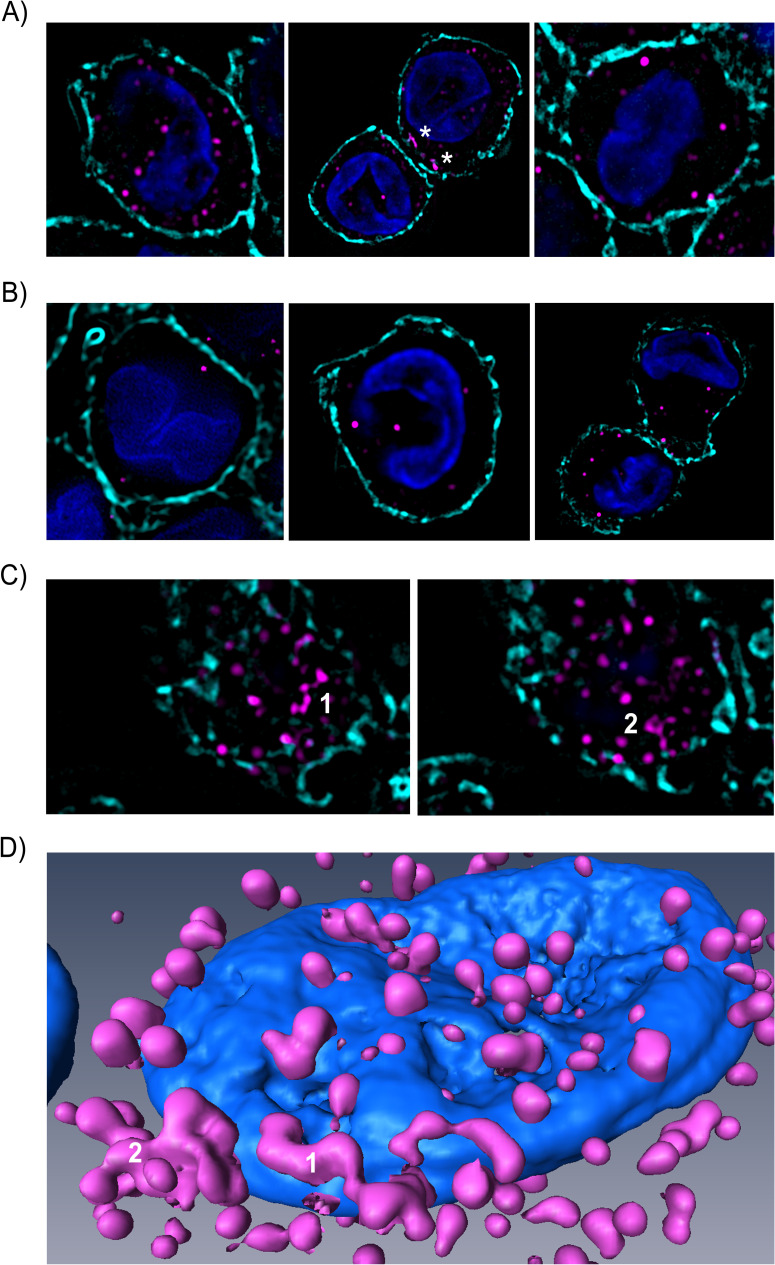
High-resolution confocal images of genotype Ia BKPyV particles endocytosed in MDDCs. Three panels showing independent cells that contain intracellular dot-like or amorphous tube-shaped (white asterisk) accumulations of Alexa Fluor 647-conjugated gIa VLPs (A) or BKPyV infectious particles (B; Dunlop strain). Images show focal planes extracted from six different cells stacks (10^4^ VLPs/cell or 1FFU/cell respectively for VLPs and infectious particles; magenta). (C) Two distinct focal planes extracted from the cell stack from which the image in the center of Fig 2A is shown. “1” and “2” indicate the tube-shaped structures marked by asterisks in Fig 2A. (D) Amira 3D reconstruction of the cell represented in Fig 2A (center) showing round-shaped and pleiomorphous tube-shaped intracellular structures containing Alexa Fluor 647-conjugated gIa VLPs. Cell membranes were stained with fluorescence-labelled WGA (Alexa Fluor 488 displayed in light blue) and nuclei were counterstained with DAPI. High-resolution confocal images were obtained from the A1 Nikon microscope equipped with a SIM module.

**Fig 3:**
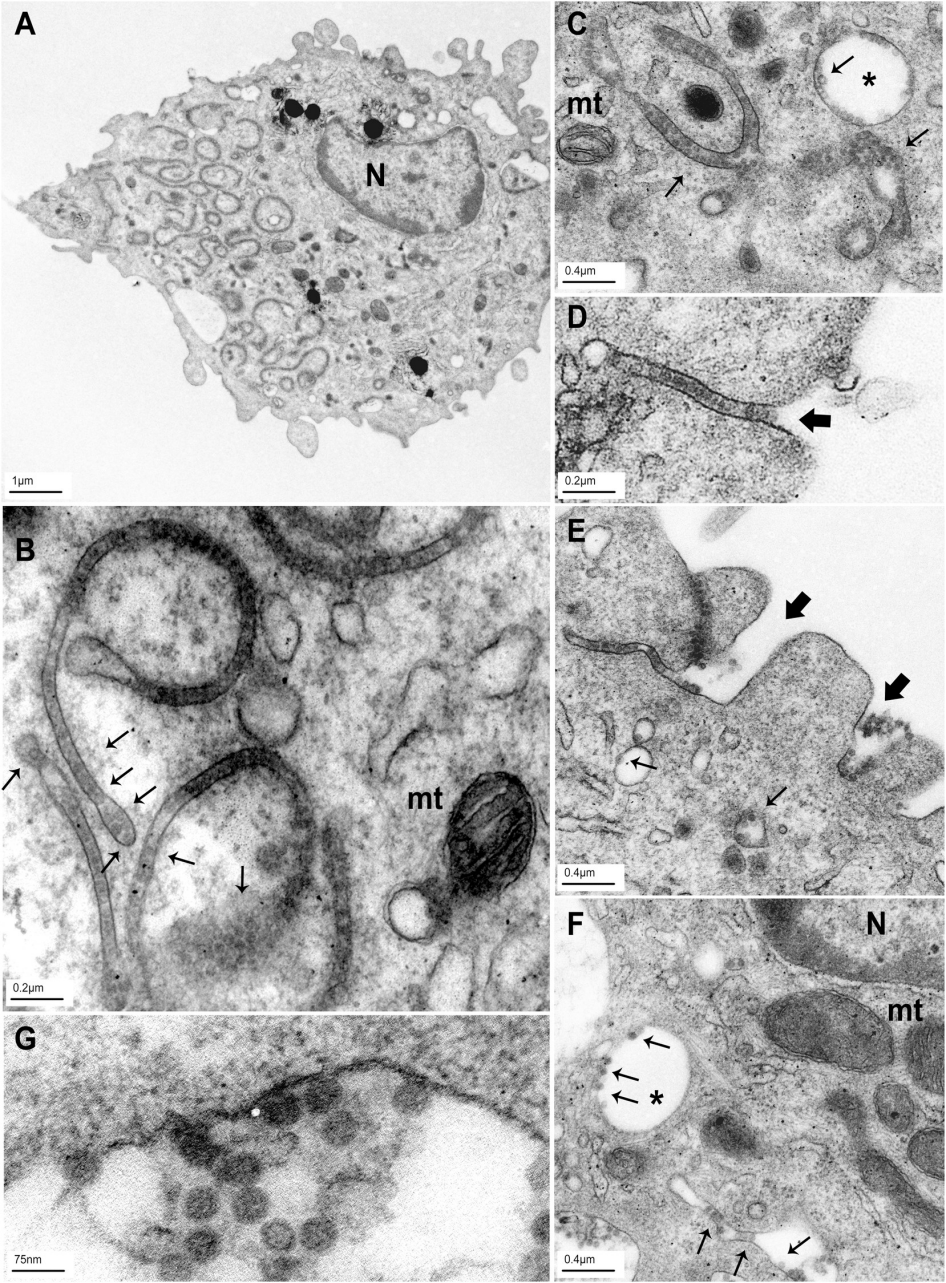
TEM reveals unlabeled BKPyV endocytosis into large round- and tube-shaped vesicles in MDDCs. (A) Micrograph showing a general view of a representative MDDC incubated for 30 minutes at 37°C with 10^4^ VLPs/cell (gIb2). Noticeably, the cell contains abundant tube-shaped structures. (B, C, D, E and G) Pleiomorphous or large round vesicles containing VLPs are shown at a higher magnification (G: x100,000–120,000). (F) This image represents infectious BKPyV particles into macropinosome-like (round-shaped) and tube-shaped vesicles (1FFU/cell). (D) and (E) Micrographs showing VLP internalization from the cell surface into tube-shaped endosomes. Thin and bold arrows indicate particles and tube formation respectively; asterisk indicate large vesicles resembling macropinosomes. N = nucleus; mt = mitochondria. Scale bars are indicated for each micrograph.

Immature MDDCs exhibit high endocytic properties for soluble and particulate Ags[41]. Therefore, we hypothesized that BKPyV could be endocytosed following attachment to sialic acid residues on PM. High-resolution confocal imaging showed that fluorescent spots representing VLPs or virions were found in cytoplasmic structures (Fig 2A and 2B), confirming that MDDCs endocytosed BKPyV following surface attachment. VLPs were either located in round-shaped or pleiomorphous tubular structures (Fig 2A and 2C). This was confirmed by 3D cell reconstruction (Fig 2D). Then, we performed TEM imaging and confirmed that VLPs and virions were mostly internalized after 30 minutes. Indeed VLPs were mainly endocytosed into tubular vesicles (40-60nm width) and to a much lower extent in large round-shaped uncoated endosomes (up to approximately 1μm in diameter) by MDDCs (Fig 3A, 3B, 3C, 3D, 3E and 3G). Moreover, these BKPyV-containing tubular vesicles were shown to originate from PM invaginations (Fig 3D and 3E). Some of these vesicles closely resembled sorting endosomes (Fig 3C). Higher magnifications micrographs confirmed that BKPyV virions behaved similarly to VLPs (Fig 3F) did not reveal PM curvature upon viral attachment as previously reported for SV40 (Fig 3G; [42]). We concluded that BKPyV was mainly endocytosed into tubular vesicles evoking an uncommon endocytic pathway for viral particles in MDDCs.

### BKPyV colocalizes with GRAF-1^+^ and cholera toxin B^+^ compartments in MDDCs

**Fig 4:**
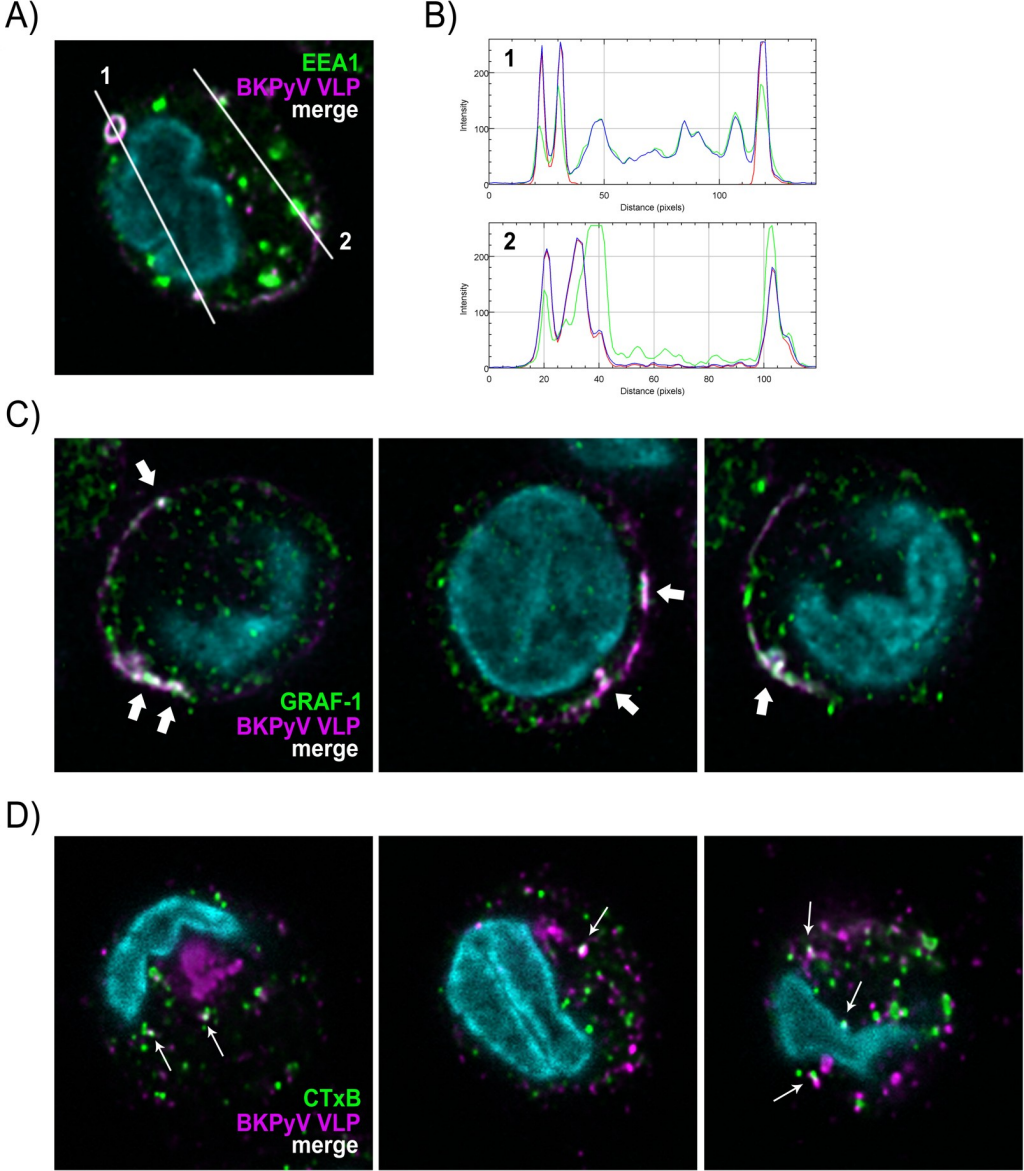
BKPyV particles colocalize with EEA-1, GRAF-1 and CTxB in MDDCs revealing an unconventional endocytic pathway. (A) Confocal sections of MDDCs incubated for 30 minutes at 37°C with 10^4^ fluorescent VLPs/cell (magenta). EEA-1-positive endocytic vesicles were stained after fixation (green). Nuclei were counterstained with DAPI (light blue). The colocalization between VLPs and EEA-1 is shown in white. (B) RGB profiles along two measurement lines (1 and 2, showed in Fig 3A) analyzed with ImageJ software. Colocalization is represented by merging blue and red (= magenta) representing VLPs and green histograms (1 pixel = 88nm). (C) and (D) show respectively colocalization of BKPyV VLPs with GRAF-1 (bold white arrows) and Alexa Fluor 555-conjugated cholera toxin subunit B (CTxB; 2μg/mL; thin white arrows). Deconvoluted images are presented. Displayed data are representative of three independent experiments.

To characterize BKPyV containing vesicles in MDDCs we used high-resolution confocal microscopy to identify markers co-localizing with BKPyV in MDDCs. Early Endosome Antigen-1 (EEA-1), a marker of early endosomes and macropinosomes, was associated with BKPyV in structures with size ranging from 100nm, the detection limit with this technique, to roughly 1μm in diameter (Fig 4A and 4B). The clathrin-independent carriers (CLIC) or GPI-anchored protein-enriched compartments (GEEC) endocytic pathway[43,44] known to form tubular vesicles has been recently associated with the protein GTPase Regulator Associated with Focal Adhesion Kinase-1 (GRAF1)[45]. BKPyV colocalized with GRAF-1 at the PM and in the cytosol (Fig 4C). Cholera toxin B subunit (CTxB) uses GRAF-1 vesicles to enter cells[45] and we observed a partial VLP/CTxB colocalization in MDDCs (Fig 4D). Altogether, our results showed for the first time in MDDCs a major BKPyV endocytosis into GRAF-1^+^ and CTxB^+^ compartments, two hallmarks of the CLIC/GEEC pathway.

### MDDCs can transfer virions to renal epithelial cells but are refractory to BKPyV infection

**Fig 5:**
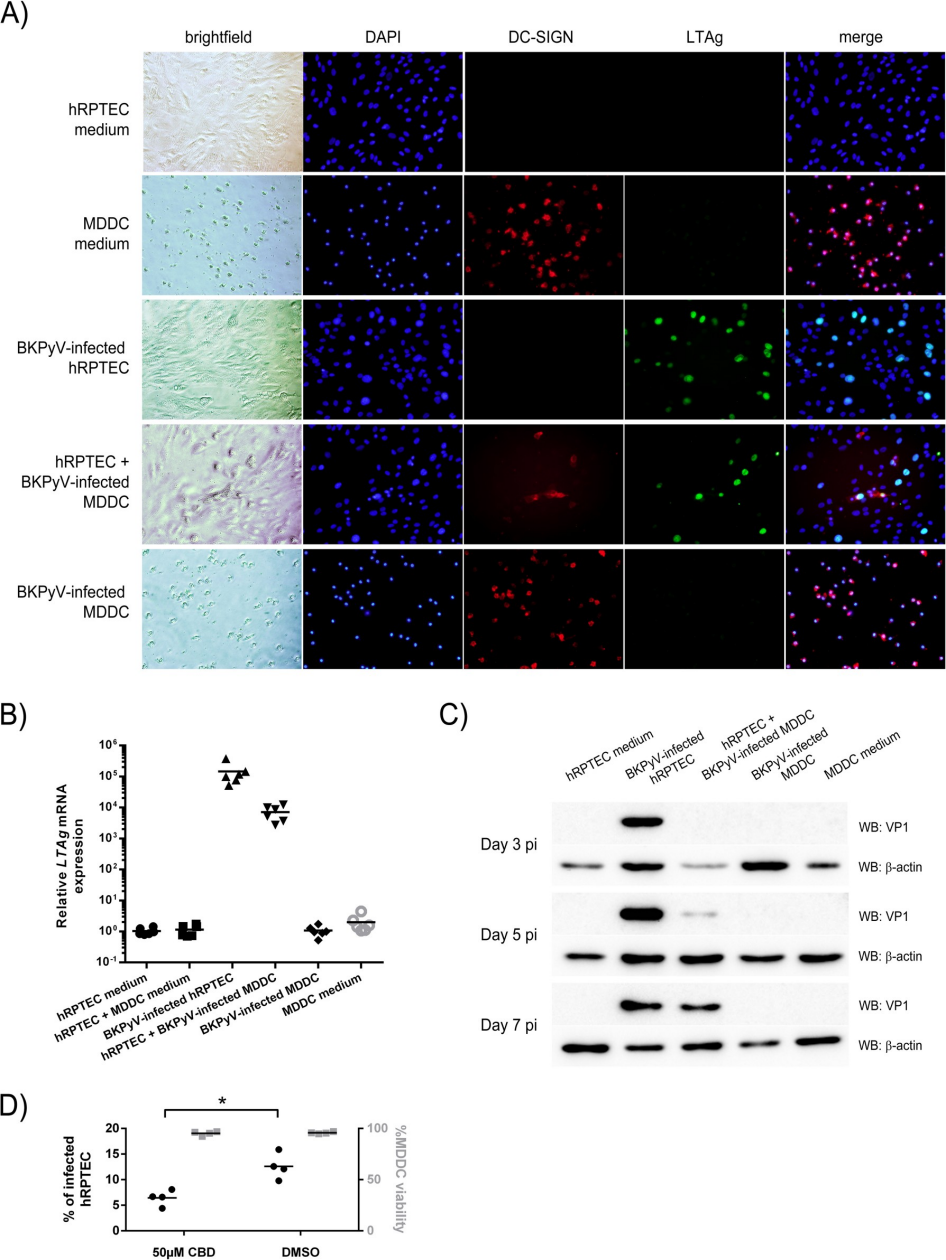
MDDCs do not support BKPyV infection but mediate its transmission to primary hRPTECs. (A) Epifluorescence microscope images (x10 magnification) showing large T antigen (LTAg) immunostaining (green) of hRPTECs and/or MDDCs in various conditions indicated on top of rows, respectively: hRPTECs alone (medium), MDDCs alone (medium), BKPyV-infected hRPTECs (MOI = 0.1; approximately 200 particles/cell), non-infected hRPTECs layered with BKPyV-infected MDDCs (excess of virus, i.e. unbound virus, was removed by extensive washes after a 2 hour-incubation of MDDCs with virus) and BKPyV-infected MDDCs (*idem* previous condition). LTAg is revealed at seven dpi. Brightfield images of the immunostaining are shown in the first column. DC-SIGN (red) is a marker allowing to discriminate MDDCs from hRPTECs when necessary. Nuclei were counterstained with DAPI. (B) RT-qPCR data showing the amplification of LTAg mRNA seven dpi in various conditions (similar to those presented in Fig 6A; n = 6). Of note, a condition with uninfected MDDCs with uninfected hRPTECs has been added here. (C) Western blot analysis of VP1 expression, as a late BKPyV infection event, in cell lysates after three, five, and seven dpi. β actin was revealed similarly after membrane stripping as a loading control. Fig 5A and 5C are representative of three independent experiments.

Next, we wondered whether BKPyV-pulsed MDDCs, hereafter termed “BKPyV-infected MDDCs”, could transfer the virus to a permissive cellular third party in *trans*. Here, we took advantage of a *trans*-infection assay previously set up in our laboratory[46,47]. It assesses the ability of a cell type to capture and transfer virions to permissive cells in its vicinity after removing excess unbound/non-internalized virions. LTAg expression was analyzed in these conditions at defined time points (Fig 5A). Infection of hRPTECs, termed *cis*-infection, was estimated between 10 to 18% in all experiments at seven dpi (Fig 5A). No LTAg was detected in BKPyV-infected MDDCs suggesting that BKPyV infection is not initiated in MDDCs (Fig 5A). An apparent DC-SIGN/LTAg colocalization event observed for one cell in the “BKPyV-infected MDDCs” condition was attributed to an optical illusion due to the superimposition of two cells, one expressing DC-SIGN and an underlying LTAg^+^ hRPTEC. The caption of the Fig 5A indicates that these pictures were obtained with an epifluorescence microscope explaining such a rare artefactual event. To confirm these results with a more sensitive technique, we analyzed *LTAg* expression by RT-qPCR in a similar experimental design. Quantitative results are shown in Fig 5B. As expected, no LTAg mRNA was detected in BKPyV-infected MDDCs whereas the *cis*-infection of hRPTECs or the *trans*-infection conditions displayed high amounts of *LTAg* mRNA. These results were confirmed by assessing the expression of the major capsid protein VP1, a late infection marker (Fig 5C). To confirm the CLIC/GEEC pathway involvement in the BKPyV *trans*-infection process, we finally tested the effect of the ciliobrevin D (CBD), a cytoplasmic dynein inhibitor[48], on MDDCs during virus loading. Noticeably, a 50μM dose of CBD significantly decreased *trans*-infection with no measurable MDDC cytotoxicity (Fig 5D). Altogether, these results demonstrated that MDDCs, while non-permissive to BKPyV, capture BKPyV virions and can transfer them to permissive cells like hRPTECs in a dynein-dependent manner.

### Human MDDCs are neither activated by BKPyV particles nor BKPyV-infected hRPTECs

**Fig 6:**
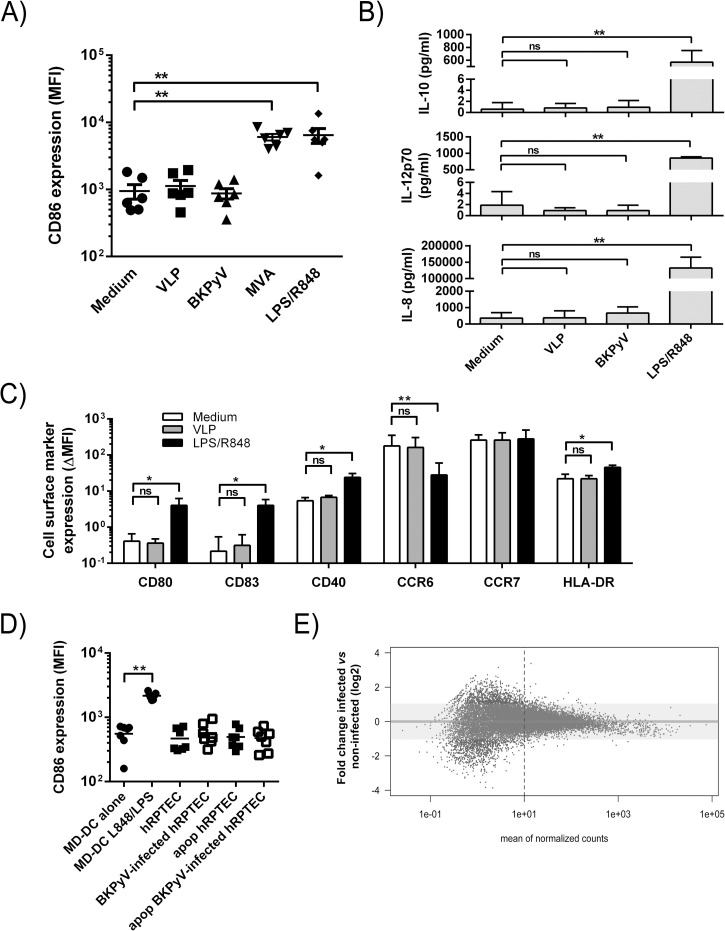
BKPyV virions or BKPyV-infected cells fail to activate MDDCs. (A) CD86 cell surface expression was assessed by flow cytometry on immature MDDCs alone (circles) or cultured with VLPs (squares; 10^3^ particles/cell), BKPyV particles (triangles; 10^3^ particles/cell) MVA (inverted triangles) or a TLR agonist cocktail (diamonds; 100ng/mL LPS and 1μg/mL R848 after 24 hours (n = 6). (B) ELISA titration of IL-10, IL-12p70 and IL-8 in the supernatants of untreated or MDDCs cultivated with VLPs, BKPyV particles or R848/LPS (doses were similar to those employed in Fig 4A). (C) Cell surface expression of CD80, CD83, CD40, CCR6, CCR7 and the HLA-DR on MDDCs alone (empty bars) or cultured for 24 hours with VLPs (grey bars; 10^3^ particles/cell) or LPS/R848 (black bars). Data are represented as MFI ± SEM. For each MFI, background, i.e. autofluorescence, is subtracted to calculate ΔMFI values displayed in this figure (n = 4). (D) Similar to experiments in A. Apop = apoptotic cells. Apoptosis was induced by UVB-irradiation and apoptotic cell fragments were collected by centrifugation and extensive washing in PBS. (E) RNAseq analysis of differentially expressed genes between infected (one dpi) and non-infected MDDCs. The dashed line represents a “ten counts per gene” limit above which gene expression is considered as robust. The Y axis represents the Log2 fold change in gene expression. Statistically significant results were marked by one or several asterisks according to the level of significance: ns = non-significant, * = p<0.05, ** = p<0.01, *** = p<0.001, **** = p<0.0001; one-way ANOVA with Tukey’s multiple comparison tests.

MDDCs can sense danger signals through various pattern-recognition receptors (PRR) including toll-like receptors (TLRs) thus leading to MDDC maturation[49,50]. Conflicting results on DC activation by BKPyV in the literature prompted us to ask whether BKPyV attachment would lead to MDDC activation. Twenty-four hour MDDC cultures with VLPs or virions were analyzed by flow cytometry to assess the acquisition or up-regulation of known DC maturation markers. A maturation enabling dose of LPS and R848[51], two TLR agonists, and a Modified Vaccinia Ankara (MVA) attenuated poxvirus known to activate MDDCs were added as positive maturation controls. First, expression of CD86, a sensitive and reliable marker of DC maturation[52], was assessed. Only exposure to TLR agonists or to the MVA known to activate MDDC-induced CD86 upregulation ([53–56]; Fig 6A). Accordingly, no IL-12p70, a T helper type 1 cytokine, IL-10 or IL-8 were detected in MDDC culture supernatants cultivated with BKPyV (Fig 6B). Expression of the CD80, CD83, CD40, CCR7 and HLA-DR on MDDCs gave consistent results (Fig 6C). Then we hypothesized that MDDCs could be activated not by BKPyV particles *per se* but by BKPyV-infected hRPTECs. HRPTEC infection was monitored by RT-qPCR for *LTAg* mRNA expression as already shown in Fig 5B. In that setting, MDDC CD86 expression did not vary upon cultivation with BKPyV-infected cells (Fig 6D). To exclude “under the radar” activation signals, we finally performed a digital RNA sequencing (DGEseq; [57]) of BKPyV-infected MDDCs compared to non-infected cells. In line with previous experiments, no difference was observed between BKPyV-infected and non-infected MDDCs in terms of mRNA profile reprogramming at one dpi (Fig 6E). These results confirmed that MDDCs were unresponsive to BKPyV and BKPyV-infected hRPTECs.

### Internalized BKPyV is protected from neutralization

**Fig 7:**
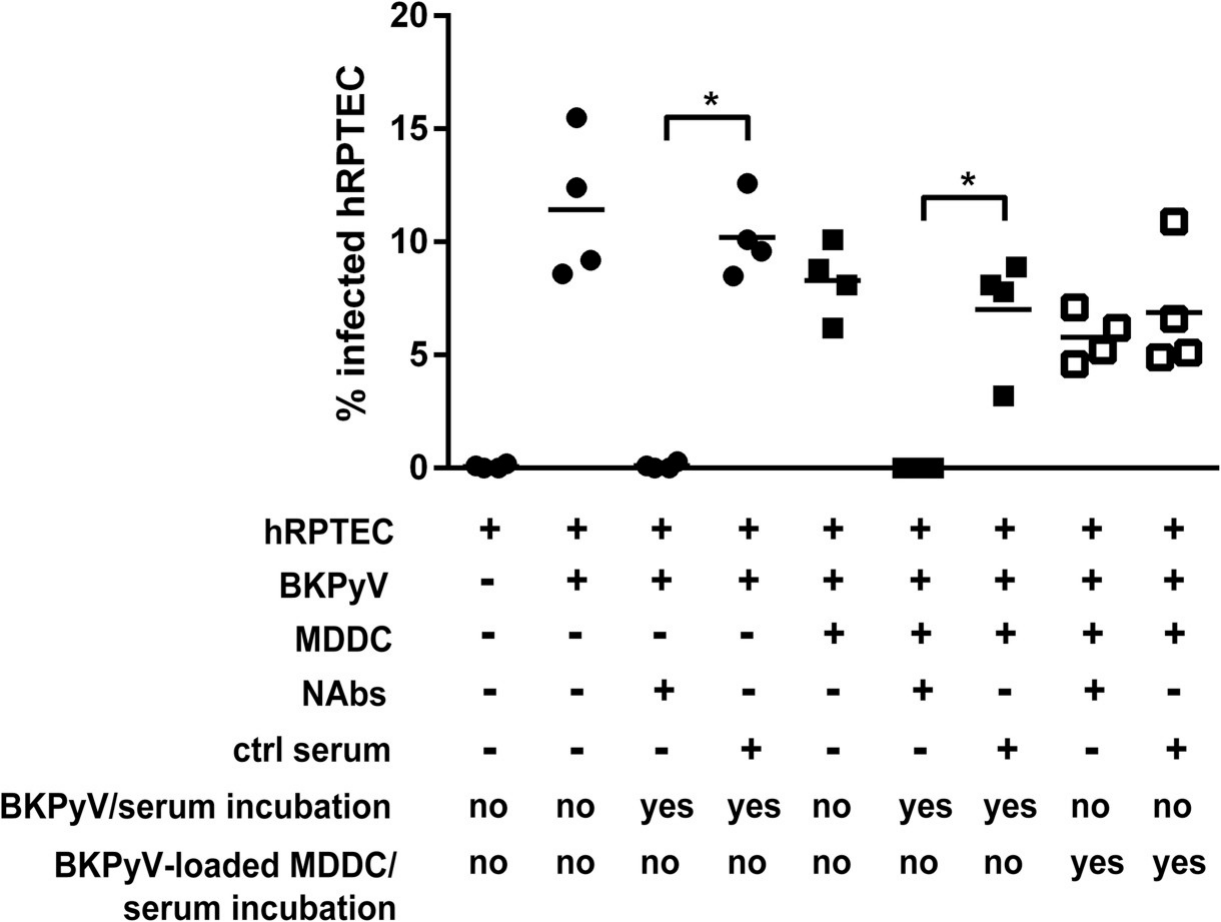
Endocytosed BKPyV particles into MDDCs are protected from serum neutralization. HCS automated counting to evaluate percentages of BKPyV-infected hRPTECs (= LTAg^+^ cells) in various conditions including *cis*- and *trans*-infection experiments but with or without sera from a seronegative patient (= control serum, neutralization titres <1/200) or a controller patient (= neutralizing serum). Neutralizing antibody titers in this serum had been previously determined: between 1/200.000, 1/500.000 and 1/20.000 for genotypes Ia, Ib2 and IVc2 respectively (see *Cis* and *Trans*-infection assays in Methods). Here, both sera were x1000-diluted. Sera were either added before or after incubation of the BKPyV suspension (Dunlop strain at MOI = 0.1) with MDDCs. Results represent mean values of the percentage of LTAg^+^ hRPTECs ± SEM. Statistically significant results are marked by an asterisk; * = p<0.05; one-way ANOVA with Tukey’s multiple comparison tests.

Together with cellular immune responses, neutralizing anti-BKPyV antibodies (NAbs) are required to control infection or reactivation in KTRs[13, 19, 58–60] and healthy donors[61]. Here we wondered whether BKPyV could be protected from neutralization when internalized by MDDCs. To address this point, *trans*-infection was performed in the presence of neutralizing and control sera from BKPyV reactivating or non-reactivating KTRs respectively. Neutralizing antibodies completely blocked hRPTEC *cis*-infection whereas the control serum had no effect (Fig 7). When virions were pre-incubated with NAbs prior to MDDC loading, a significant loss in *trans*-infection was observed compared to controls. As a conclusion, NAbs were ineffective when used after BKPyV loading of MDDCs suggesting virions were protected from neutralization once internalized although post-attachment conformation changes of the viral capsid dampening *trans*-infection cannot be ruled out at this stage.

### Blood and kidney CD1c^+^ DCs display similar BKPyV *trans*-infection abilities and non-permissiveness to MDDCs

**Fig 8:**
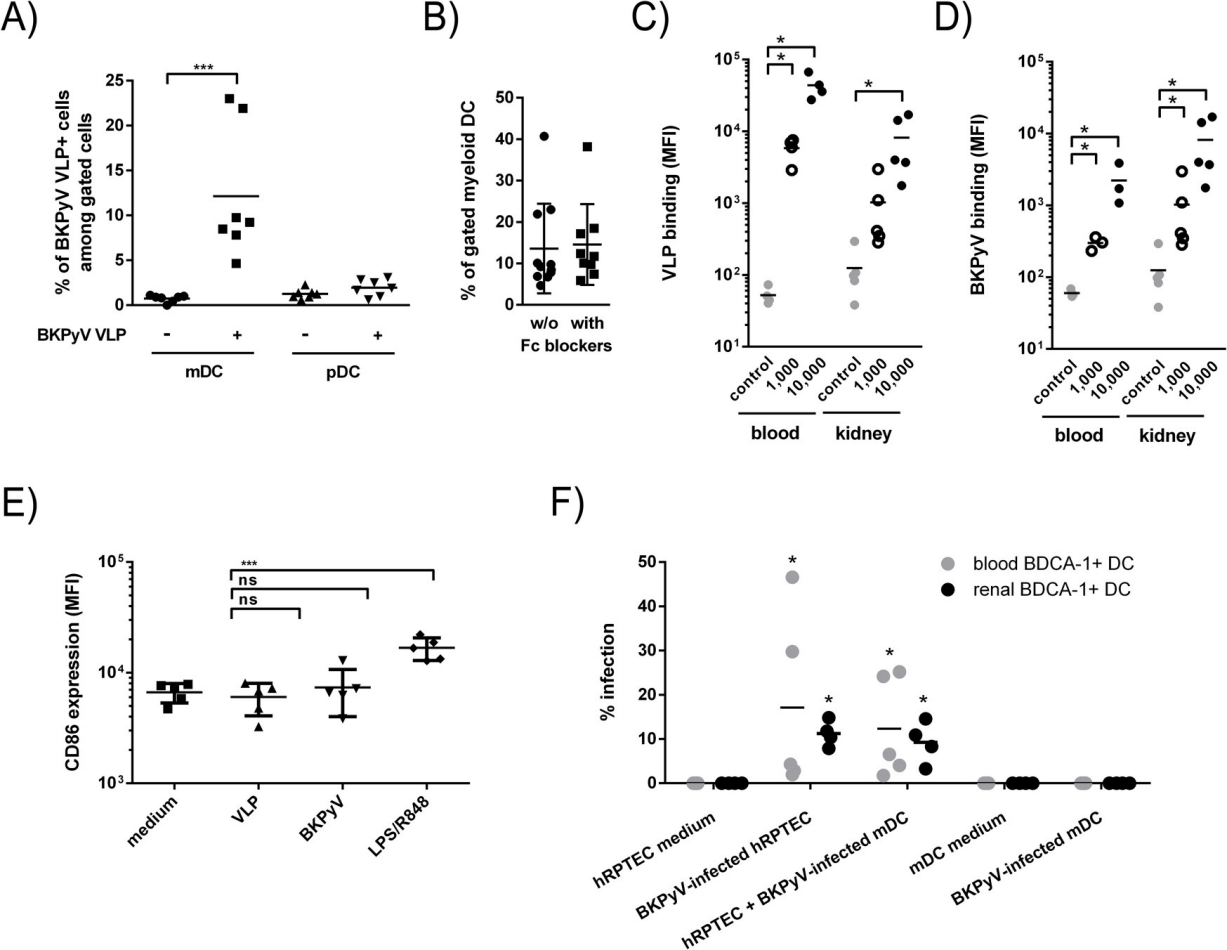
Blood and kidney CD1c^+^ myeloid DCs bind and transmit BKPyV to primary hRPTECs without getting infected. (A) Quantitative measurement of VLP positive cDCs and pDCs in whole blood of healthy volunteers according to the gating strategy shown in S1 Fig. (B) Quantitative analysis of VLP binding to cDCs with (closed squares) or without (closed circles) Fc receptor blockade. Dose-dependent BKPyV VLP (C) or infectious particle (D) binding to purified CD1c^+^ DCs from blood or kidney of healthy individuals; the “control” condition means no VLP (grey circles). Black closed and empty circles represent 10^3^ and 10^4^ particles/cell respectively. The immunomagnetic cell sorting strategy is shown in S1 Fig. (E) CD86 cell surface expression assessed by flow cytometry on freshly isolated blood CD1c^+^ DCs cultured for 24 hours in medium alone (squares) or with VLPs (triangles; 10^3^ particles per cells), BKPyV particles (inverted triangles; ibid) or with LPS/R848 (diamonds; 100ng/mL LPS and 1μg/mL R848); n = 4 distinct blood donors. (F) Quantitative assessment of the ability of CD1c^+^ mDCs from blood (grey dots) or kidney (black dots), ie from healthy blood donors and macroscopically healthy parts of resected human tumor-bearing kidneys, to capture and transfer BKPyV to hRPTECs as shown in Fig 6A. The percentage of infected hRPTECs corresponds to the percentage of LTAg^+^ hRPTECs within total cells, i.e., DAPI counterstained nuclei(47), based on an automated counting on a HCS device. Data are represented as percentage of infection. Statistical analyses have been applied to comparisons between % of infected hRPTECs in coculture with cDCs with or without preincubation with the Dunlop strain. Statistically significant results are marked by an asterisk; * = p<0.05, ** = p<0.01, *** = p<0.001; one-way ANOVA with Tukey’s multiple comparison tests.

MDDCs were shown to be closely related to inflammatory DCs in humans[62,63] so to ensure our observations were not biased by the DC generation protocol, we first wondered whether cDCs, the most abundant tissue and blood DC subset under non-inflammatory conditions, could behave like MDDCs. First, VLPs were incubated with whole blood of healthy volunteers and VLP staining was further analyzed by flow cytometry on both cDCs (CD11c^+^) and plasmacytoid (pDCs; CD123^+^) DCs among HLA-DR^+^ Lin^-^ cells (S1 Fig). A significant proportion of cDCs bound VLPs whereas no binding was detected on pDCs (Figs 8A and S1). Importantly, binding to cDCs was not affected by Fc receptor blockade suggesting that VLP attachment did not depend on anti-VP1 antibodies in whole blood of healthy donors (Fig 8B). To avoid any interference due to the whole blood environment, CD1c^+^ DCs, representing the main myeloid DC subset in blood[64] and kidney[65] were sorted according to the gating strategy displayed in S2 Fig, incubated with VLPs and analyzed by flow cytometry. Blood and kidney CD1c^+^ DCs were clearly capable of binding VLPs as well as virions in a dose-dependent manner (Fig 8C and 8D, respectively). Then, we showed that like MDDCs, CD1c^+^ DCs were unresponsive to BKPyV particles (Fig 8E). We finally demonstrated that sorted CD1c^+^ DCs enabled BKPyV *trans*-infection to permissive cells while being resistant to infection themselves (Fig 8F). Taken together our results demonstrate that biologically relevant blood and renal CD1c^+^ DCs behave similarly to MDDCs with respect to BKPyV infection.

## Discussion

In this study, cDCs, either generated *in vitro* or isolated from human blood and kidney, were shown to support BKPyV attachment in a sialic acid-dependent manner and subsequent clathrin-independent endocytosis through two distinct pathways, the first involving GRAF-1^+^ CLIC/GEEC and the second, minor pathway dependent on EEA1^+^ macropinocytic endocytosis. However, we did not provide evidence on potential spatio-temporal connections between both compartments. Upon contact, viral particles or BKPyV-infected cells failed to activate cDCs. Moreover, we showed that MDDCs and CD1c+ DCs were non-permissive to BKPyV infection. Internalized or membrane-bound BKPyV virions kept their ability to *trans*-infect permissive cells like hRPTECs and were also shown to be protected from neutralization by sera of BKPyV reactivating KTRs.

We demonstrated that BKPyV interacts with human MDDCs in a dose- and sialic acid-dependent manner suggesting these cells are equipped with BKPyV receptors, likely GD1b and GT1b[39]. Upon attachment, BKPyV accumulates massively in pleiomorphous GRAF-1^+^ endocytic vesicles originating from the PM and partially overlapping with CTxB containing vesicles in MDDCs. Although not proven here, it is tempting to speculate that a similar entry pathway for BKPyV occurs in CD1c-sorted cDCs. This compartment was identified as CLIC/GEEC vesicles whose formation is clathrin-independent (see for review [66]). Interestingly, BKPyV was shown to infect hRPTECs in a clathrin- and caveolin-independent manner indicating that some BKPyV entry steps might be common between those cells and cDCs[67,68]. Ewers and colleagues demonstrated that SV40 and CTxB interactions with long acyl chain-containing GM1 in caveolin-1 deficient or energy-depleted cells triggered the formation of PM invaginations related to CLIC/GEEC endocytosis[42]. Multiple interactions between chemically defined GM1 PM clusters and SV40 were demonstrated to promote PM curvature and the formation of pleiomorphous tubules containing viral particles. A previous report by Stergiou et al. brought evidence that the SV40 infection is dependent on the GRAF-1-mediated inactivation of RhoA in the human A431 cell line[69]. Although this study focused on a role of GRAF-1 in intracellular signaling events, it is tempting to speculate that, like BKPyV, SV40 may utilize a GRAF-1-dependent endocytosis as a general entry route in various cell types. In this study, the ganglioside-deficient GM95 cell line supports SV40 infection in a α2β1 integrin-dependent manner suggesting GM1 is dispensable. However, much effort is needed to address this point comprehensively. CLIC/GEEC endocytosis has not been thoroughly documented for BKPyV before our results, even though Drachenberg et al. observed BKPyV virions within tubular structures in hRPTECs from PVAN biopsies and suggested that this could be a way for BKPyV to reach the nucleus[32]. Although the microtubule-associated motor protein, dynein 1, was not shown to play a role in BKPyV[70], JCPyV and SV40 infection[71], here we demonstrated that a specific chemical inhibitor of the dynein protein family caused a measurable reduction of the *trans*-infection process by MDDCs indicating distinct cell-specific requirements for BKPyV entry. After internalization, BKPyV[72], JCPyV[72,73], SV40[72,74] and MuPyV[75] were shown to reach the ER within the first ten hours after cell attachment to hRPTECs[39,70]. This step is crucial for infection[39,70,76,77] since it is followed by the release of partially uncoated virions in the cytosol and import of viral genomes to the nucleus to initiate replication. This question remained unaddressed in the present study but the absence of LTAg in MDDCs after several dpi supports several hypotheses. First, a cell-intrinsic or virally induced defect between endocytosis and release of the viral genome into the nucleus, including the uncoating step, is possible. In line with this assumption, we have been unable to detect uncoated virions in the cytoplasm of MDDCs by TEM and no attempt was made to seek for viral episomes in their nucleus. If the viral genome can reach the nucleus of myeloid DCs, which remains to be demonstrated yet, a potential negative regulation of the viral transcription may be caused by the lack of appropriate transcription factors and/or repressors in those cells compared to permissive cells. This was previously shown for MCPyV[78]. Moreover, Bethge and colleagues nicely reported the importance of the Sp1 transcription factor (TF) to regulate the transcription of early and late BKPyV genes[79]. Sp1 is ubiquitous in human DCs but other TFs may be involved. We demonstrated in this study that BKPyV entry into DCs occurs mainly through the CLIC/GEEC endocytosis but also through a macropinocytosis-like pathway although the latter was much rarely observed. One could imagine that the CLIC/GEEC pathway may lead to a non-productive infection in DCs though CLIC/GEEC was reported as the main productive AAV2 infection route in non-immune cells[80]. These discrepancies between our results and former studies might reflect multiple common as well as distinct BKPyV entry steps according to the cell type studied. Therefore, further work is needed to establish the molecular determinants of such differences in infecting non-immune and immune cells like cDCs with BKPyV. A recent review pointed out the link between the CLIC/GEEC endocytosis and glycosphingolipids (GSLs) which encompass gangliosides in the establishment of cell polarity[66]. DC polarization leading to the formation of a synapse is an important event in T cell priming (see for review [81]) but might also be crucial in the BKPyV *trans*-infection process we described here. GSLs are known to form lipid rafts on the PM (see for review [82]). Such micro domains function as a platform to segregate a wide range of effector molecules including GPI-anchored cargos[83]. Wang and colleagues demonstrated that GPI-anchored molecules, which utilize the CLIC/GEEC endocytic pathway upon ligand binding, share biosynthetic pathways and common cellular locations with GSLs[84]. Such findings could link the ganglioside-mediated BKPyV attachment to viral endocytosis even in immune cells.

An important DC function is the ability to sense microbes through the recognition of conserved pathogen-associated molecular patterns (PAMPs) by PM-bound (toll-like and C-type lectin receptors, TLRs and CLRs respectively), endosomal (TLRs) or modified DNA/RNA cytoplasmic receptors altogether termed PRRs[85]. Viral particles as supramolecular arrangements of proteins and nucleic acids can function as PAMPs. Zepeda-Cervantes et al. have recently discussed numerous examples of VLP sensing leading to activation of human DCs in a review[86]. In contrast, both *in vitro*-generated murine and human DCs were shown to remain unresponsive to BK- and JCPyV VLPs[25,28]. Our results with human MDDCs as well as freshly isolated blood and renal CD1c+ DCs confirm these observations and extend them to *bona fide* DC subsets supporting that such an immune ignorance towards BKPyV could exist *in vivo*. Two recent comprehensive studies demonstrated that hRPTECs fail to sense BKPyV[87,88]. This was shown to be partly dependent on the expression of the agnoprotein, a viral factor whose function has remained unclear so far[88]. While BKPyV escape mechanisms seem to depend on viral gene expression in hRPTECs, we consider that different escape mechanisms are at work in BKPyV refractory cDCs. The observed accumulation of BKPyV into CLIC/GEEC vesicles in MDDCs after two hours might lead to their segregation in a PRR-free compartment. Unfortunately, whether PRRs are present in the CLIC/GEEC compartment is unknown.

We demonstrated that although renal CD1c+ DCs are refractory to BKPyV infection they remain able to capture virions and *trans*-infect hRPTECs *in vitro*. CD1c^+^ DCs are normally present in the human renal interstitium surrounding the proximal tubules and glomeruli[31,36,65] but in PVAN lesions, a significant increase in infiltrating CD1c+ DCs is documented[36]. Whether the CD1c^+^ DC infiltrate has a key role in viral spreading *in vivo* deserves deeper investigations with combined multidimensional imaging techniques and spatial RNA/DNA sequencing. Upon inflammation, monocytes are recruited in tissues where they differentiate in inflammatory DCs with transcriptomic profiles closely related to those observed in MDDCs[62,63]. PVAN develops in an inflammatory context. Therefore, it is tempting to speculate that along with resident CD1c^+^ DCs, inflammatory DCs could participate in the potentiation of BKPyV infection. All these observations provided the rationale for investigating interactions between BKPyV and MDDCs or CD1c^+^ DCs reported here. We showed that MDDCs and CD1c+ DC were capable of internalizing viral Ags while remaining in an immature state thus. These results could indicate an impossibility for those cells to properly process and present BKPyV-derived Ags to effector T lymphocytes. Another myeloid DC subset expressing the CD141 marker exists in humans. Alternatively named cDC1, those cells cross-present viral Ags very efficiently through the indirect pathway, i.e., the engulfment of virally-infected necrotic cells and presentation of pMHC I complexes to CTLs[89]. An elegant study recently performed by Silvin and colleagues demonstrated that CD1c^+^ DC were permissive to enveloped viruses like HIV-1 or Flu inducing their death thus providing viral Ags to CD141^+^ DCs which were shown to be resistant to infection by these pathogens[90]. Whether such a functional specialization of myeloid DC subsets exists for BKPyV infection has now to be determined.

In this study, we demonstrated that cDCs, namely MDDCs and blood or kidney CD1c^+^ resident DCs could capture infectious BKPyV through an unprecedented endocytic pathway in cDCs and for BKPyV, and transfer the virus to permissive cells like hRPTECs without DC activation or infection, suggesting a role for cDCs in BKPyV spreading. Moreover, we showed that internalized virions were protected from neutralization by serum from KTRs. Taken together our results support the idea that cDCs could facilitate BKPyV infection by favoring its spreading and limiting specific T lymphocyte activation due to the cDC ignorance towards BKPyV antigens and the circumvention of neutralization by specific antibodies. Hence, this work could help to understand how cDCs could aggravate BKPyV infection in KTRs.

## Methods

### Ethic statements

Biopsies from healthy parts of primitive renal carcinoma patients and blood samples from KTRs were collected according to institutional guidelines (CPP Ouest authorization, 11/08/2011) and under patients’ formal consent, verbal or written. All samples are conserved in the ITUN bio collection declared at the french Ministère de l’Enseignement Supérieur et de la Recherche under the reference DC-2011-1399 (09/05/2011).

### Cell isolation and culture

Elutriated blood monocytes were obtained from healthy volunteers (DTC cell-sorting facility, CHU Nantes, France) and differentiated into MDDCs as described by Sallusto et al.[41]. Human myeloid CD1c^+^ DCs were isolated from blood and kidney by positive immuno-magnetic selection using anti-CD1c/BDCA-1 microbeads according to the manufacturer’s instructions (Miltenyi Biotec, Bergisch Gladbach, Germany) or on a FACS ARIA (BD Biosciences, Franklin Lakes, NJ), respectively. CD1c^+^ DC were recovered from renal cell suspensions of enzymatically digested macroscopically healthy parts of tumor-bearing kidneys (10-15g). Cell purity typically yielded more than 95%. HRPTECs (Sciencell Research Laboratories, Carlsbad, CA) were cultured in complete EpiCM medium (Sciencell Research Laboratories). LNCaP cells (Caliper LifeSciences, Hopkinton, MA) and HEK 293 TT cells (NCI, Frederick, MD) were cultured in RPMI 1640 or DMEM media respectively, both complemented with 2mM L-glutamine and 10% FBS.

### Virus and virus-like particle preparation

The BKPyV Dunlop strain was a kind gift by Dr Christine H Rinaldo (UiT, Norway). The gIa, gIb2 and gIVb1 VP1 expression vectors were kindly provided by Dr Christopher Buck (NCI, USA)[91]. Preparation and titration of the Dunlop strain were performed as described elsewhere[92]. VLPs were purified on an iodixanol gradient[93]. VLP physical titers were determined on a qNano device using NP100 nanopores (detection range from 50 to 330 nm) and CPC70 calibration particles (Izon Science Ltd, Oxford, UK). Both viral particles and VLPs were labelled with Alexa Fluor 647 protein labelling kit according to manufacturer’s instructions (Molecular Probes, Eugene, OR). Modified-vaccinia Ankara virus (MVA) was kindly provided by Pr Don Diamon (CoH, Los Angeles, CA).

### *Cis* and *Trans*-infection assays

*Cis*- and *trans*-infection experiments were performed as described previously[47, 94]. DCs and hRPTECs were infected with BKPyV at MOI 0.1 (Dunlop strain). For trans-infection, BKPyV-loaded DCs were washed in PBS after two hours at 37°C then put in contact with a subconfluent hRPTEC monolayer. Controls were prepared similarly. Neutralization of *trans*-infection was achieved using cells incubated with sera from a seronegative patient (= control serum, neutralization titres <1/200) or a controller patient who developed high neutralizing antibody titres in response to BKPyV viremia (= neutralizing serum from a BKPyV reactivating KTRs). Neutralizing antibody titers in this serum had been previously determined: between 1/200.000, 1/500.000 and 1/20.000 for genotypes Ia, Ib2 and IVc2 respectively. Both sera were x1000-diluted. After three to seven days post-infection (dpi), LTAg staining was performed to evaluate infection rates as described before[92] and imaged on an Axiovert A1 epifluorescence microscope (Carl Zeiss Microscopy GmbH, Germany) or on a Cellomics ArrayScan VTI HCS Reader (Thermo Scientific) for quantification. 25–50 fields, containing 5000–10000 cells were acquired for each well using HCS Studio Cellomics Scan Version 6.5.0 software at various time points post-infection (pi). VP1 expression was assessed by western blot (ab53977; Abcam) against β-actin (clone C4; Santa Cruz Biotechnology Inc., Dallas, Texas).

### Quantitative RT-PCR analyses

Total RNA was isolated using the TRIzol reagent (Invitrogen) according to the manufacturers’ instructions. Reverse transcription was performed using M-MLV Reverse Transcriptase and random primers following manufacturer’s instructions (Invitrogen, USA). Quantitative PCR on reverse transcribed mRNA was performed using Mastermix (Applied Biosystems) or Premix ExTaq 2x (Takara) reagents and the StepOne Plus (Applied Biosystems) or Rotor-Gene (Qiagen) devices. Primers and probe used to detect *LTAg* mRNA were the following: AgT1 5’-ACTCCCACTCTTCTGTTCCATAGG-3’, AgT2 5’-TCATCAGCCTGATTTTGGAACCT-3’ and AGTS 5’-FAM-TTGGCACCTCTGAGCTAC-BHQ1-3’. Expression levels were normalized to GAPDH using the 2-ΔΔ cycle threshold method.

### Gene expression profiling and datasets deposition

BKPyV-mediated cell reprogramming was analysed after 24 hours by 3′digital gene expression (DGE) RNAseq according to Kilens et al. [57]. DGE profiles were generated by counting for each sample the number of unique UMIs associated with each RefSeq genes. DESeq 2 was used to normalize expression with the DESeq function. The analysis design used to perform differential expression with DESeq2 between the infected *vs* non-infected conditions took into account the individual DC donors as a confounding variable. Data supporting our results are openly available in the GEO repository under the following ID: GSE154810.

### Flow cytometry analyses

Titrated Alexa Fluor 647 labelled-VLP stocks were used to stain cDCs, LNCaP and HEK 293 TT cells at the indicated concentration. VLP attachment was detected by flow cytometry gated on DAPI negative cells. To assess DC activation, cells were incubated for 24 hours with 10^3^ VLPs/cell, 10^3^ BKPyV particles/cell or with 100ng/mL LPS and 1μg/mL R848 (Invivogen, San Diego, CA). Antibodies to CD40 (clone 5C3; BD Biosciences), CD80 (clone L307, BD Biosciences), CD83 (clone HB15e, BD Biosciences), CD86 (clone IT2.2, BD Biosciences), CCR6 (clone 11A9, BD Biosciences), CCR7 (clone 3D12, BD Biosciences) and HLA-DR (clone G46-6, BD Biosciences) were used to monitor DC maturation. Whole blood staining was performed on 500μl blood samples from healthy donors with or without Fc fragment receptor blockers (Miltenyi Biotec). Whole blood staining was done with Alexa Fluor 647 labelled-VLPs (2.5μg/ml) and cell subsets were discriminated using the following antibody panel: CD45 (Clone J33; Beckman Coulter, Brea, CA), CD11c (Clone BU15; Beckman Coulter), HLA-DR (Clone L243; BD Biosciences), CD123 (Clone 9F5; BD Biosciences) and Lineage (Lin 1; BD Biosciences). FACS analyses were mainly performed on a LSR II flow cytometer (BD Biosciences).

### Fluorescence microscopy

MDDCs were distinguished from hRPTECs by DC-SIGN staining (clone DCN46; BD Biosciences) when required. High-resolution confocal microscopy by structured illumination was performed to assess BKPyV entry into MDDCs. Cells were incubated with determined VLP concentrations for one hour in culture medium to allow for VLP internalization at a physiological temperature, 37°C, washed and fixed with 3.7% PFA to freeze cellular structures (PFA; Electron Microscopy Sciences, Hatfield, PA). PM was stained with 5μg/mL Alexa Fluor 488-conjugated WGA (Thermo Fisher Scientific). Images were acquired on a Nikon N-SIM microscope with a dedicated oil immersion objective (x100, NA 1.49 Plan Apo). Three dimensional optical sectioning was done respecting Nyquist sampling rate (15 structure illuminations per plane, per channel), and super resolution image reconstruction was performed using Nikon Imaging Software algorithms. BKPyV particle colocalization with CTxB, GRAF-1 and EEA-1 markers was performed as described above with or without 2μg/mL Alexa Fluor 555 conjugated CTxB (Thermo Fisher Scientific), and with anti-GRAF1 (4μg/mL; Novus Biological, Littleton, CO) or anti-EEA1 antibody (BD Biosciences) antibodies in 0.1% BSA PBS O/N at 4°C. Nuclei were counterstained with DAPI. Cells were mounted in ProLong mounting medium (Thermo Fisher Scientific) and observed on a LSM Nikon A1RSi microscope (Nikon, Tokyo, Japan) at x60 (NA 1.4). 3D reconstruction was done using the Imaris software (Bitplane, Zurich, Switzerland).

### Transmission electron microscopy

MDDCs were prepared for transmission electron microscopy as described elsewhere[94]. Ultrathin sections were observed on a JEM 1010 microscope (Jeol Europe SAS, Croissy Sur Seine, France). TEM images of BKPyV particle preparations in negative contrast were obtained as described previously[95,96].

### ELISA

Supernatants from various MDDC cultures were harvested at indicated times and frozen at -80°C until being analyzed. IL-10 and IL-12p70 were quantified in those culture supernatants by ELISA with BD OptEIA human IL-10 and IL-12p70 sets following the manufacturer’s instructions (BD Bioscience).

### Statistics

Statistical analyses were performed with the PRISM software (GraphPad Software Inc., version 5.04, La Jolla, CA). Almost exclusively one-way ANOVA with multiple comparison tests were performed to assess significance in this study. Exceptionally, correlation and linear regression studies, Mann-Whitney or Friedman tests were also applied to some data sets. *P*-values lower than 0.05 were considered significant. All numerical values used to build up figures in this manuscript are gathered in S1 Data.

## Supporting information

S1 Fig Gating strategy of myeloid (CD11c^+^ in CD45^+^, HLA-DR^+^, Lin^-^ cells) and plasmacytoid DCs (CD123^+^ in CD45^+^, HLA-DR^+^, Lin^-^ cells) in whole blood of healthy volunteers. Cells were incubated with 2.5μg/mL of Alexa Fluor 647 coupled-VLPs or the same volume of PBS (no VLP; 45 minutes at 4°C) and representative dot plots showing the percentage of VLP^+^ cells.
(TIF)

S2 Fig Assessment of purity of freshly isolated CD1c^+^ DCs from blood and kidney. (A) Gating and enrichment evaluation before and after immunomagnetic cell sorting of CD19^-^ CD1c^+^ myeloid blood DCs. (B) Similar dot plots showing the purity of CD1c^+^ myeloid DCs from resected human kidneys before and after the FACS-assisted sorting. These results are representative of five different cell isolations from both blood and kidney compartments.
(TIF)

S1 Data Excel file gathering raw data, i.e., numerical values, used to build figures showing quantitative analyses in the present study. (XLSX)
