# Supplementary figures and images for: Non-permissive human conventional CD1c+ dendritic cells enable trans-infection of human primary renal tubular epithelial cells and protect BK polyomavirus from neutralization
Source: PLoS Pathog. 2021 Feb 16;17(2):e1009042. doi: 10.1371/journal.ppat.1009042 (PMC7886149; doi:10.1371/journal.ppat.1009042)

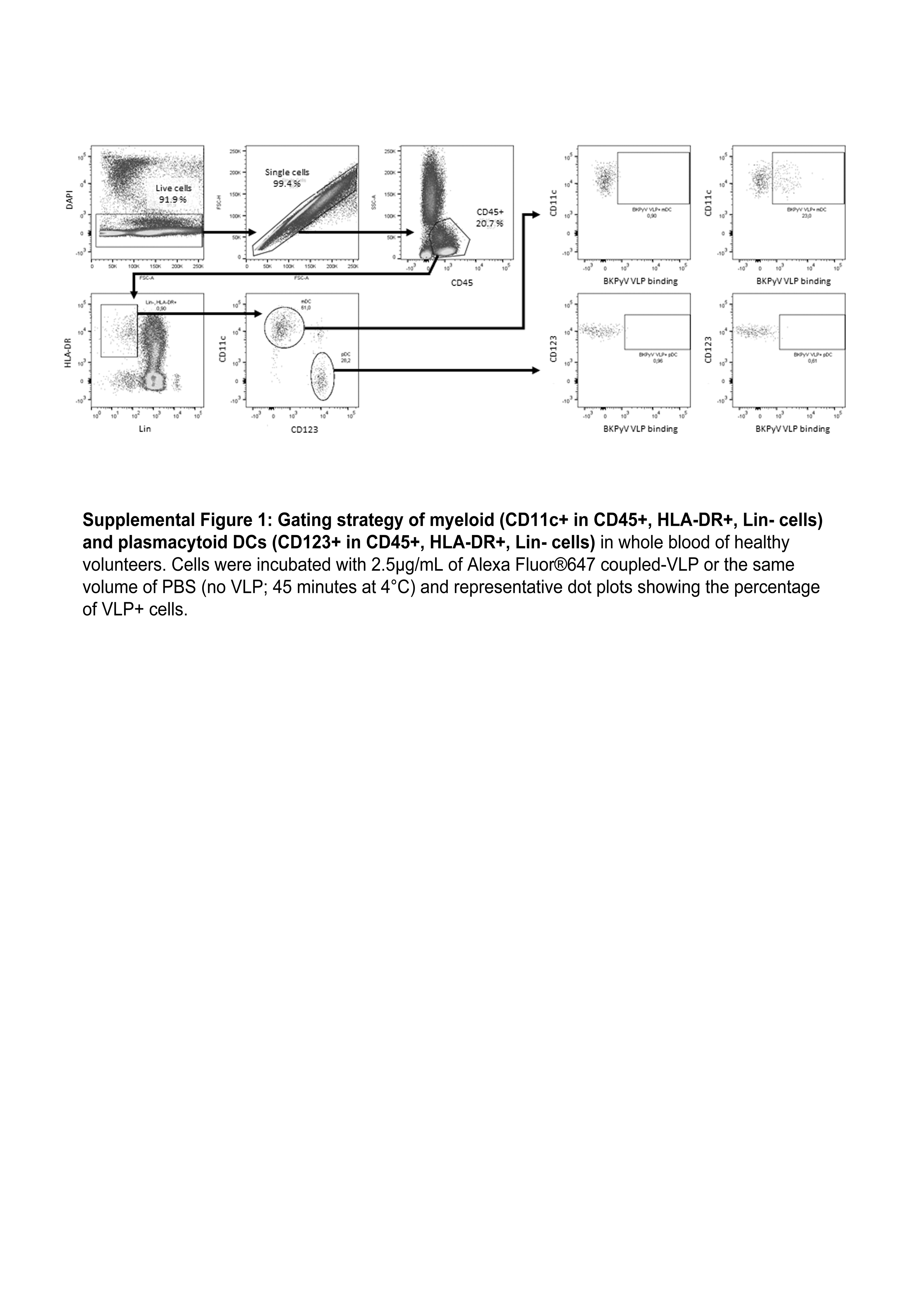

Supplement: S1 Fig — Cells were incubated with 2.5μg/mL of Alexa Fluor 647 coupled-VLPs or the same volume of PBS (no VLP; 45 minutes at 4°C) and representative dot plots showing the percentage of VLP+ cells. (TIF) [file ppat.1009042.s001.tif]

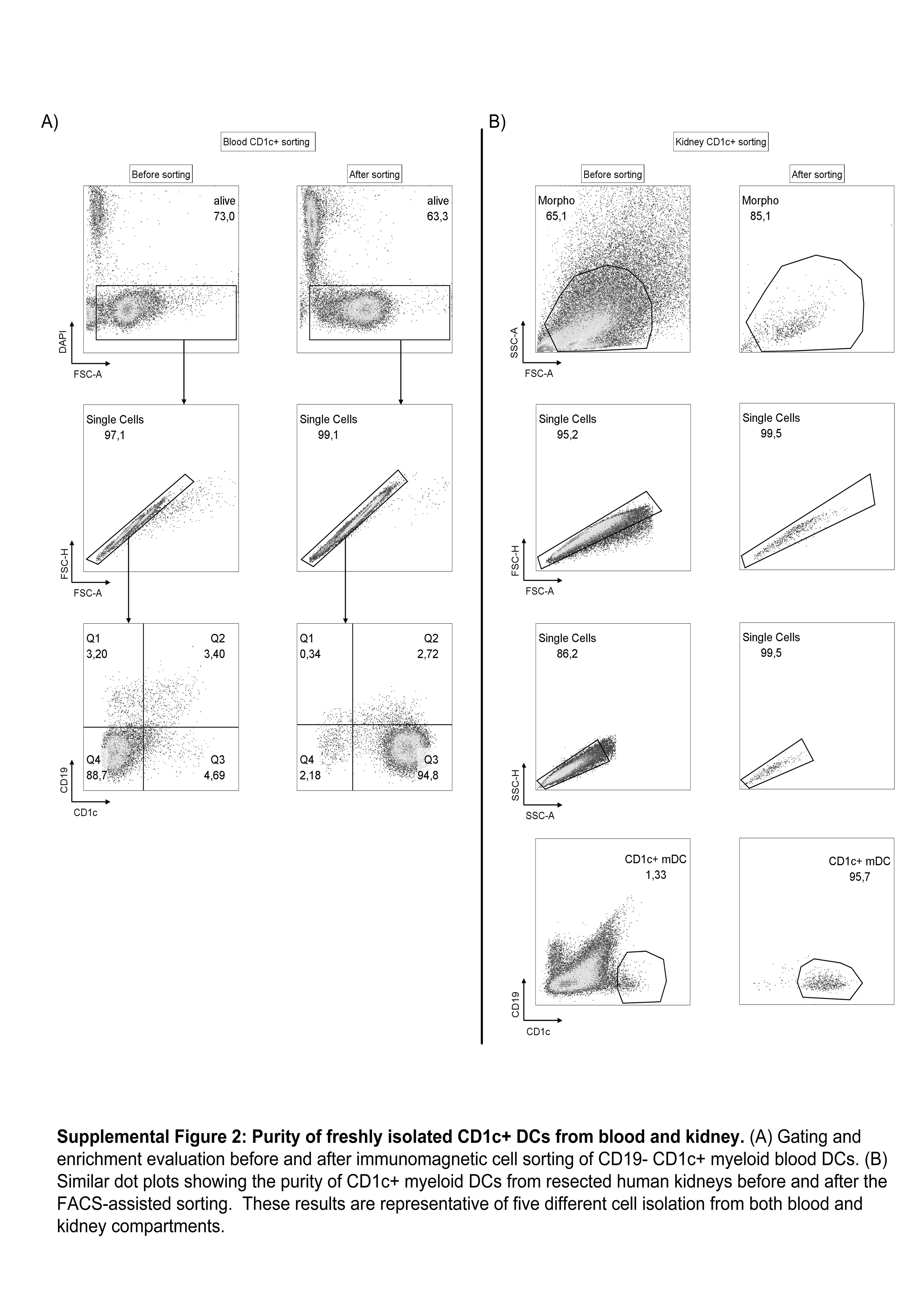

Supplement: S2 Fig — (A) Gating and enrichment evaluation before and after immunomagnetic cell sorting of CD19- CD1c+ myeloid blood DCs. (B) Similar dot plots showing the purity of CD1c+ myeloid DCs from resected human kidneys before and after the FACS-assisted sorting. These results are representative of five different cell isolations from both blood and kidney compartments. (TIF) [file ppat.1009042.s002.tif]
